# Repurposing Carvedilol as a Novel Inhibitor of the *Trypanosoma cruzi* Autophagy Flux That Affects Parasite Replication and Survival

**DOI:** 10.3389/fcimb.2021.657257

**Published:** 2021-08-12

**Authors:** Cynthia Vanesa Rivero, Santiago José Martínez, Paul Novick, Juan Agustín Cueto, Betiana Nebaí Salassa, María Cristina Vanrell, Xiaomo Li, Carlos Alberto Labriola, Luis Mariano Polo, David M. Engman, Joachim Clos, Patricia Silvia Romano

**Affiliations:** ^1^Laboratorio de Biología de Trypanosoma cruzi y la célula hospedadora - Instituto de Histología y Embriología “Dr. Mario H. Burgos”, IHEM-CONICET- Universidad Nacional de Cuyo, Mendoza, Argentina; ^2^Leishmaniasis Group, Bernhard Nocht Institute for Tropical Medicine, Hamburg, Germany; ^3^Department of Pathology and Laboratory Medicine, Cedars Sinai Medical Center, Los Angeles, CA, United States; ^4^Department of Chemistry, Stanford University, San Francisco, CA, United States; ^5^Laboratorio de Biología estructural y celular, Fundación Instituto Leloir (FIL-CONICET), Buenos Aires, Argentina; ^6^Instituto de Histología y Embriología “Dr. Mario H. Burgos”, IHEM-CONICET- Universidad Nacional de Cuyo, Mendoza, Argentina

**Keywords:** Chagas disease, *Trypanosoma cruzi*, drug repurposing, trypanocidal drugs, autophagy flux inhibitor, mice infection

## Abstract

*T. cruzi*, the causal agent of Chagas disease, is a parasite able to infect different types of host cells and to persist chronically in the tissues of human and animal hosts. These qualities and the lack of an effective treatment for the chronic stage of the disease have contributed to the durability and the spread of the disease around the world. There is an urgent necessity to find new therapies for Chagas disease. Drug repurposing is a promising and cost-saving strategy for finding new drugs for different illnesses. In this work we describe the effect of carvedilol on *T. cruzi*. This compound, selected by virtual screening, increased the accumulation of immature autophagosomes characterized by lower acidity and hydrolytic properties. As a consequence of this action, the survival of trypomastigotes and the replication of epimastigotes and amastigotes were impaired, resulting in a significant reduction of infection and parasite load. Furthermore, carvedilol reduced the whole-body parasite burden peak in infected mice. In summary, in this work we present a repurposed drug with a significant *in vitro* and *in vivo* activity against *T. cruzi*. These data in addition to other pharmacological properties make carvedilol an attractive lead for Chagas disease treatment.

## Introduction

Chagas disease is endemic to Latin America, where it is estimated to affect around 8 million people. This illness is transmitted with the feces of infected triatomine bugs, blood-sucking insects that feed on humans and animals. It can also be transmitted *via* non-vectorial routes, such as trans-placental, by blood transfusion, organ transplant and contaminated food and drinks. Lately, due to the successes in controlling domestic triatomine populations, non-vectorial routes have become the main concern. Although Chagas disease had once been restricted to Latin America, migratory movements to non-endemic areas has caused a worldwide spreading. Thus, Chagas disease is now recognized as a global health problem (Pérez-Molina and Molina, 2018).

Clinically, Chagas disease has two phases: the acute phase, frequently asymptomatic, and the chronic phase. Approximately 30 to 40% of chronically infected people will develop life-threatening cardiac or digestive abnormalities decades after the primary infection. The treatment of Chagas disease is currently limited to only two antiparasitic drugs developed in the 1970s, benznidazole and nifurtimox. In the acute phase, treatment is almost 100% effective and Chagas can be cured. However, due to its asymptomatic nature, the infection often goes undiagnosed and untreated. The available drugs present low efficacy in the chronic phase and severe side effects. Despite significant efforts over the past years to develop new drugs for Chagas therapy, none have significantly outperformed the classic treatments (Coura and De Castro, 2002; Chatelain and Konar, 2015; Kratz, 2019). Consequently, research efforts must be increased in order to find new drugs.

Chagas disease is caused by the protist parasite *Trypanosoma cruzi* which has a complex lifecycle, involving, as mentioned above, an insect vector and a vertebrate host. The parasite has different developmental stages: epimastigotes are the proliferative and presumably non-infective extracellular forms present in the insect gut. There, they differentiate into metacyclic trypomastigotes (MT), the infective forms, which are discharged with the feces during the bloodmeal of the vector. Transmission occurs when infected bug feces contaminate the bite site or mucous membranes of vertebrate hosts. Once inside the host, MTs invade a variety of cells, including macrophages, fibroblasts, and muscle cells. Here, they transform into proliferative amastigotes. Upon reaching a high intracellular parasite load, they differentiate further into bloodstream trypomastigotes, which finally reach the bloodstream by lysing the host cell.

The extensive morphological and metabolic changes underwent by *T. cruzi* during its lifecycle have been related to the induction of the autophagy pathway (Salassa and Romano, 2019). Autophagy is a catabolic process aimed at cytosolic components, such as long-lived proteins and aged or damaged organelles, for degradation by means of lysosomes. Autophagy is required during growth, development and differentiation in different organisms and is a key process to maintain cell survival during nutrient starvation (Yu et al., 2018). The process involves engulfment of a portion of cytoplasm and formation of a double membrane vesicle, called autophagosome, which subsequently fuses with lysosomes. More than 40 proteins, so-called AuTophaGy (Atg) related proteins, drive the different steps of autophagy in yeast with many of them conserved in mammalian cells (Galluzzi et al., 2017). One of them, Atg8, is widely used as an autophagy marker due its association with all autophagy structures (Klionsky et al., 2016). Similar to other eukaryotic organisms, *T. cruzi* cells possess a functional autophagy pathway which is activated in response to nutritional stress (Alvarez et al., 2008). In previous works we characterized the participation of autophagy and its modulators during *T. cruzi* metacyclogenesis, the process of differentiation of epimastigotes to MTs (Vanrell et al., 2017). We also demonstrated the key role of *T. cruzi* autophagy in cruzipain activation during parasite differentiattion and host cell infection (Losinno et al., 2020).

Cruzipain (Cz), the major cysteine protease of *T. cruzi*, has multiple roles, including its participation in the process of *T. cruzi* entry and survival into the host cell and in immune evasion (Scharfstein et al., 2000; Stempin et al., 2002; Aoki et al., 2004). These actions evidence the key role of this enzyme in the pathogenesis of *T. cruzi* infection and Chagas disease (Doyle et al., 2011) and point to Cz as a pharmaceutical target and suggest its inhibitors as leads for the treatment of Chagas disease (McKerrow et al., 2008; Ndao et al., 2014). A particularly promising cruzipain inhibitor was K777, a vinyl sulfone studied by the McKerrow laboratory (University of California San Diego, USA) which was shown to cure *T. cruzi* infection in mice and greatly reduce cardiac damage in infected dogs (Engel et al., 1998; Mckerrow et al., 2009; Doyle et al., 2011).

Similar to other neglected tropical diseases, Chagas disease remain largely overlooked by the big pharmaceutical companies as it is endemic to some of the world’s poorest regions and cannot offer the financial incentive to attract significant attention. Repurposing already approved drugs is an attractive strategy to provide new therapies for Chagas disease (Bellera et al., 2015). We found carvedilol in a virtual screening aimed at identifying compounds with potential to bind Cz. Carvedilol is a beta-blocker widely used to treat hypertension and other cardiovascular diseases, with a previously reported modulatory effect of autophagy in different kinds of cells (Meng et al., 2018; Wong et al., 2018). Preliminary data showed interesting anti-*T. cruzi* effects of carvedilol although its mechanism of action was not completely understood because it displayed not significant inhibition of Cz activity *in vitro*. Based in our previous results about the role of autophagy on Cz activation, we therefore proposed an effect of carvedilol on the autophagy response of *T. cruzi* and performed experiments to test this hypothesis. Our data confirmed that carvedilol inhibits *T. cruzi* autophagy flux and, as a result of this action, impaired parasite infection, replication and survival in host cells besides displayed a significant effect in the parasite load *in vivo*. These data highlight autophagy as a new therapeutic target in *T. cruzi* and show carvedilol as a promising lead for Chagas disease treatment.

## Materials and Methods

### Reagents

Dulbecco modified minimal essential medium (D-MEM) was obtained from Invitrogen Argentina SA (Buenos Aires, Argentina). Fetal bovine serum (FBS) was purchased from Serendipia Lab. (Buenos Aires, Argentina). The monoclonal Ab against TcAtg8.1 protein (1:500 dilution) was generously given by Dr. Vanina Alvarez (IIB-INTECH UNSAM-CONICET). The samples incubated with Ab against TcAtg8.1 were rinsed with wash solution (3 times for 10 min) and developed with Cy3-conjugated antirabbit IgG Ab (1:200 dilutions, Jackson, 111165003). The TRITC-conjugated phalloidin was purchased from Sigma (Buenos Aires, Argentina). The DNA marker Hoechst 33342 (DAPI), the Lysotracker red marker (L7526) and the DQ Red BSA (D12051) were purchased from Invitrogen Argentina SA (Buenos Aires, Argentina). The alamar- Blue reactive (DAL-1025) was purchased from Biosource-Life Technologies. Carvedilol 98% was from AK Scientific (Union City, CA, USA, 72956-09-3). Carvedlilol was diluted in Dimethyl sulfoxide (DMSO).

### Media

Diamond medium contains 6.25 g/L tryptose (Sigma, 70937), 6.25 g/L tryptone (Sigma, T7293), 6.25 g/L yeast extract (Sigma, Y1625), 7.16 g/L KH2PO4 (Biopack, 2000963500) (pH 7.2) and 6.66 mM hemin (Calbiochem, 37415GM), prepared in 3 mL 1N NaOH (Tetrahedron), and 20 mL 1 M Tris HCl (Sigma Aldrich, 10812846001), pH 6.8. BHT medium was prepared with 33 g/L Brain heart infusion broth (Britania), 3 g/L tryptose, 0.4 g/L KCl, 0.3 g/L glucose and 3.2 g/L Na2HPO4 (Biopack, 2000979000).

### Cell Culture

*T. cruzi* Y strain constitutively expressing GFP (Y-GFP) was generously provided by Dr. S. Schenkman, Universidad Federal de Sao Paulo (Sao Paulo, Brazil). Epimastigotes of Y or Y-GFP strain were cultured in Diamond medium with 10% fetal bovine serum at 28°C. All cultures contained 20 mg/L hemin (Calbiochem, 3741), 10% inactivated fetal bovine serum, 250 μg/mL geneticin (Gibco, 10131035) for GFP selection, 100 mg/mL streptomycin (Gibco, 1514022) and 100 U/mL penicillin (Gibco, 15140122). Stationary phase parasites (5 × 10^7^ cells/mL) were used in all experiments. Trypomastigotes o*f T. cruzi* Y-GFP strain were obtained by metacyclogenesis *in vitro* by using TAU and TAU-AAG medium as previously described (Barclay et al., 2011). *T. cruzi* Tulahuen strain (trypomastigote form) were generously provided by Dr. Thomas Jacobs (Bernhard Nocht Institute for Tropical Medicine, Hamburg, Germany). H9C2 (rat cardiomyoblast) cell line were grown in D-MEM medium supplemented with 10% FBS and antibiotics at 37°C in an atmosphere of 95% air and 5% CO2. HG39 (human glioblastoma line) cells were generously provided by Dr. Thomas Jacobs, (Bernhard Nocht Institute for Tropical Medicine, Hamburg, Germany), and grown in RPMI medium incubated at 37°C/5% CO2. The cells were detached using trypsin at 37° C for a few minutes. The washes were performed using 1X PBS solution (Phosphate Buffered Saline).

### Propagation of *T. cruzi*

Tissue cell trypomastigotes (TCT) of *T. cruzi* Y-GFP strain was prepared as follows. H9C2 cells (5 × 10^5^ cells/ml) were plated in T25 flasks and maintained at 37°C in D–MEM supplemented with 10% FBS and antibiotics (infection medium). Cells were infected with TCT suspensions (5 × 10^6^ cells/ml) for 3 days in infection medium at 37°C in an atmosphere of 95% air and 5% CO2. After 4 to 6 days, intracellular TCT lysed the cells and reached the medium. Medium containing parasites was harvested and centrifuged at 600 g for 15min at room temperature. The supernatant was discarded, and the pellet, containing TCT and amastigotes, was covered with 1ml of fresh medium and incubated for 3 h at 37°C to allow TCT to swim up. Supernatant enriched in TCTs was harvested, and parasites were counted in a Neubauer chamber and used for infection assays. Trypomastigotes of the *T. cruzi* Tulahuen strain were prepared in cultures of HG39 cells and maintained in RPMI medium supplemented with 10% FBS and antibiotics at 37°C using the same protocol than *T. cruzi* Y-GFP strain. All procedures involved live *T. cruzi* were made under a biosafety level II and approved by the institutional biosecurity committee.

### Transmission Electron Microscopy

Epimastigotes were exposed to 10 μM carvedilol during 24 h, 48 h and 10 days at 28°C before fixation while control parasites were incubated with the same volume of vehicle (DMSO) than treated parasites during the same time. Both samples were fixed for 1h with 2% glutaraldehyde (Ted Pella), diluted in 0.1 M cacodylate buffer in PBS for 2 h at 4°C, washed three times with PBS, pH 7.2, and subsequently treated with 1% osmium tetroxide for 2 h at 4°C. In a next step, parasites were washed again with PBS and sequentially dehydrated in solutions with increasing concentrations of acetone solutions (50%, 70%, 90%, and two exchanges of 100% acetone) for 10 min each. Finally, samples were cast in epoxy resin (Spurr), and ultrathin sections were prepared in an ultramicrotome Leica Ultracut R. Sections were contrasted with 5% uranyl acetate/acetone for 3 min, washed with distilled water and colored with lead citrate for 2 min before observation in a Zeiss 900 electron microscope.

### Indirect Immunofluorescence

The TcAtg8.1 protein was detected in epimastigotes of *T. cruzi* Y strain by indirect immunofluorescence. Parasites, previously treated in the absence (DMSO) or the presence of 10 µM carvedilol in Diamond (control) or starvation medium (Stv) for 2 h, were fixed with 4% paraformaldehyde, washed 3 times for 10 min with PBS, incubated in 50 mM NH4Cl in PBS 30 min, rinsed 3 times for 10 min with 0.05% saponin, 0.2% BSA in PBS (wash solution), and incubated overnight at 4°C with the monoclonal Ab against TcAtg8.1 protein (1:500 dilution). Samples were then rinsed with wash solution (3 × 10 min) and developed with Cy3-conjugated antirabbit IgG Ab (1:200 dilutions, Jackson,111165003). Stained parasites were mounted on coverslips with Mowiol 4–88 reagent (Calbiochem) and examined by confocal microscopy in an Olympus FV 1000 confocal microscope (Olympus, Japan). Autophagy response was analyzed in each condition by quantification of the percentage of parasites with more than two TcAtg8.1 positive vesicles.

### Fluorescence Microscopy

For detection of hydrolytic compartments, Y-GFP strain epimastigotes previously treated in the absence (DMSO) or the presence of 10 µM carvedilol in Diamond (control) or starvation medium (Stv) for 24 h, were incubated with 10 μg/ml of DQ-BSA for the last 40 min, washed three times with PBS and then mounted on coverslips with Mowiol before examination. This compound emitted red fluorescence after BSA hydrolysis into small peptides in lysosomes, thus identifying lytic compartments. Data were represented using the mean values of the percentage of DQ-BSA positive parasites (parasites with more than 2 DQ-BSA positive vesicles) ± SE, observed in a confocal microscope (Olympus FV 1000). For Lysotracker studies, epimastigotes previously treated in the absence (DMSO) or the presence of 10 µM carvedilol in Diamond (control) or starvation medium (Stv) for 24 h, were incubated with 10 μg/ml of Lysotracker Red for the last 2 h, washed three times with PBS and then mounted on coverslips with Mowiol before examination in a confocal microscope (Olympus FV 1000). All parasites stained with Lysostracker were counted and expressed as percentage of Lysotracker positive parasites.

### Effect of Carvedilol on Axenic Cultures of Epimastigotes

Epimastigotes (1×10^7^ parasites/ml) of *T. cruzi* Y-GFP strain were incubated in Diamond medium in the presence (10 µM) or the absence (only the vehicle, DMSO) of carvedilol. The number of parasites in each condition were quantified every 48 h in a Neubauer chamber.

### Cell Infection Assays

H9C2 cells (rat cardiomyoblast cell line) were infected with trypomastigotes of *T. cruzi* Y-GFP strain with a MOI of 10 for 24 h, followed by a chase of 48 h in the absence (DMSO) or the presence of 2.5 µM, 5 µM and 10 µM carvedilol. Infected cells were fixed with 4% paraformaldehyde in PBS for 15 min at room temperature, washed with PBS, and quenched with 50 mM NH_4_Cl for 15 min at room temperature. Samples were incubated with Rhodamine-phalloidine probe for 2 h to label host cell actin microfilaments, and then were mounted with Mowiol. Quantification of intracellular amastigotes in each condition was done from images obtained by confocal microscopy, using an Olympus Confocal FV1000 microscope. Data were processed using the FV10-ASW 1.7 software (Olympus).

### Vitality tests of host cells

The Alamar Blue test that measures mitochondrial activity was used to evaluate the toxicity of carvedilol on host cell cultures. Cells were incubated in 3% DMEM in 96 wells plates in the absence (DMSO) or the presence of 2.5, 5 and 10 μM carvedilol for 48 h. They were then incubated with 3% DMEM medium in the presence of 10% Alamar Blue for 6 h at 37°C and mitochondrial activity was measured by spectrofluorometry (excitation wavelength 530-560 nm, the emission wavelength 590 nm).

### Opera Phenix System to Evaluate Growth of Intracellular Amastigotes

The Perkin Elmer Opera Phenix™ System was used for phenotypic screening. For this system, 60,000 HG39 cells were plated on 96-well plates. The cells were infected with *T. cruzi* Tulahuen (MOI = 10) for 24 hours, followed by washing and subsequent treatment with carvedilol (10 µM) in RPMI medium for 24, 48 or 72 h, with daily medium change. Control cells were maintained in RPMI medium plus an equal volume of carvedilol vehicle (DMSO). Cells were fixed with 4% paraformaldehyde, and immune staining was performed with mouse anti-Leishmania HSP90 serum (Ommen et al., 2010) (1: 5000) and Alexa Fluor647 anti-mouse (1: 8000) + DAPI (1: 100). The numbers of parasites under each condition were quantified in the Opera Phenix System (Bea, 2002).

### Semi-Quantitative Real-Time PCR (qPCR) of Genomic DNA (gDNA)

A dual-labeled qPCR to analyze the parasite load of *T. cruzi* on infected cells was performed to detect parasite actin gene DNA relative to the host cell actin gene DNA as described previously (Bifeld et al., 2016). Cells were infected with *T. cruzi* Tulahuen (MOI = 10) for 24 hours, followed by washing and subsequent treatment with carvedilol (10 µM) in RPMI medium for 24, 48 or 72 h, with daily medium change. Control cells were maintained in RPMI medium plus vehicle (DMSO). At specific times, genomic DNA was purified from control and infected cells using the ISOLATEII Genomic DNA Kit (Bioline GmbH, Germany) following the manufacturer’s instructions. The qPCR master mix was prepared following the manufacturer’s manual (Sensifast Probe No-ROX kit, Bioline). The concentrations of the applied primers and probes were adjusted to the particular infection system. 10% template volume of the final reaction volume was applied to the master mix. The TaqMan probe-based qPCR allows a high gene specificity by using target gene-specific probes which anneal to the amplified region. The probe is labelled with a fluorochrome at the 3′ end and a quencher at the 5′end. The fluorochromes send fluorescence signals subsequent to the probe degradation by the Taq DNA polymerase during the PCR elongation step. The duplex qPCR was performed with host cell actin- and *T. cruzi* actin gene-specific primer sets. The primers used were: *T. cruzi* AcF 5’CGTGAGAAGATGACACAG3’; *T. cruzi* AcR 5’GGGAGAGAGTATCCCTCG3’; *T. cruzi* AcProbe FAM-5’CACGCCATCACCAGCATCAAG3’-BHQ-1; Human HuAcB-F2 5’CCCATCTACGAGGGGTATG3’; HuAcB-R2 5’GCGCTCGGTGAGGATCTTC3’; HuAcB-Probe2 CY5- 5’CCTGGCTGGCCGGGACCTGAC3’BHQ-3. The qPCR was performed as a one-step PCR using the Rotor-Gene 6.1.81™ Instrument with the setting Enzyme activation 95°C 5 min Hold, Denaturation 95°C, 10 s, annealing/extension/data acquisition 66-57.5°C 40s, 37 cycles, then Hold 2 40°C 30 sec.

### Tunel Assay

The Roche TUNEL staining kit for flow cytometry, generously provided by Dr. Lidia Bosurgi (Bernhard Nocht Institute for Tropical Medicine, Hamburg, Germany), was used to analyze the possible induction of programed cell death (PCD) on trypomastigotes after carvedilol treatment. We purified trypomastigotes from cultures by the swim-up standard procedure. Briefly, TCT of *T. cruzi* Tulahuen strain contained in the supernatants of culture medium was harvested and centrifuged at 600 g for 15 min at room temperature. The supernatant was discarded, and the pellet, containing TCT and amastigotes, was covered with 1 ml of fresh medium and incubated for 3 h at 37°C to allow motile trypomastigotes to distribute. Supernatant enriched in TCTs were incubated in RPMI in the presence of 10 µM carvedilol or DMSO (untreated control) during 24 h. Other samples of parasites were treated with DNAse and used as a negative control or heated to 90°C for 5 minutes and used as positive control. The manufacturer’s protocol was followed and measured in FACS LSRII.

### Animal Ethics

All experiments using animals were conducted according to the Guide for the Care and Use of Laboratory Animals and specifically reviewed and approved by the animal care and use committee (ACUC007053) in Cedars-Sinai Medical Center, Los Angeles, California, USA. All animals were purchased from Jackson Laboratory and were maintained under pathogen free conditions and 12 h dark/light cycle at a temperature 22 + 3°C. They had access to food and water *ad líbitum*. Female mice aged 8-12 weeks were used in all experiments. All mice were euthanized under deep anesthesia at the end of the experiment or when showing pain symptoms.

### Mice Infection Protocol

Bioluminescent *Trypanosoma cruzi* Tulahuen (discrete typing unit: TcVI) (Luc-mNeonGreen) were generated as the same methods described by (Lewis et al., 2018). The bioluminescent *Trypanosoma cruzi* Tulahuen strain trypomastigotes expressing dual reporter gene Luc-mNeonGreen previously described obtained from H9c2 cell culture were using for a SCID mice infection (5000 parasites) by intraperitoneal (IP) and monitoring the bioluminescence intensity every 3-5 days. Mice were injected by IP with 150 mg/kg d-luciferin (GOLDBIO^®^), then anaesthetized using 3% (vol/vol) gaseous isoflurane with oxygen. The luciferase intensity was measured using an IVIS Spectrum (Caliper life science) and analyzed with Softmas Pro. Exposure time varied between 5 sec. to 60 sec., depending on signal intensity. Once detected a high intensity of parasitemia (1x10^8^ per ml of blood), blood from SCID mice were harvested from the saphenous vein and washed with PBS 1X.

Healthy adults (8-12-week age) female Balb/c mice were used for drugs treatment infected initially with 5000 trypomastigotes by IP injection. Mice were treated with oral gavage doses of carvedilol 25 mg/kg/day (Sigma-Aldrich^®^) powder dissolve in a 2% methyl-cellulose (Sigma-Aldrich^®^) solution with sterilized water and administered with disposable sterile flexible teflon gavage needles (able scientific™). An equal volume of 2% methyl-cellulose (Sigma-Aldrich^®^) solution with sterilized water was administered to control animals by oral gavage. The bioluminescence intensity measure previously described was determined every 3, 4 and 7 days at the 45 DPI.

### Statistics

Data are presented as mean values and error bars indicate the SEM from at least three independent experiments. One-way ANOVA statistical analysis was performed with Tukey multiple comparison test by using Kyplot statistical software. Significance levels established by p-values: *p < 0.05; **p < 0.01; ***p < 0.001.

## Results

### Screening of Approved Drugs and Selection of a Possible Anti-*T. cruzi* Drug

Compounds that inhibit Cz activity are good candidates for Chagas disease treatment. Therefore, to identify a new Cz inhibitor, we performed an *in silico* screen against the SWEETLEAD library of approved drugs (Novick et al., 2013). Cruzipain inhibitor K777 was used as a query ligand. The conformation of K777 was extracted from the X-ray crystal structure of cruzipain in complex with this drug. We used ROCS software to perform the selection of compounds (ROCS 3.0.0 OpenEye Scientific Software, Santa Fe, NM. http://www.eyesopen.com); (Hawkins et al., 2007).

**Figure 1:**
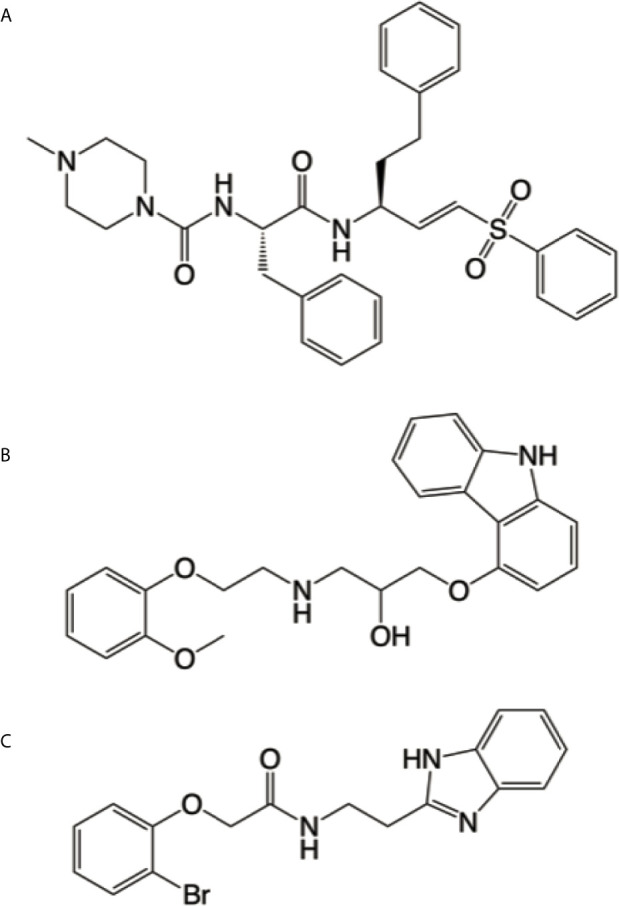
Cruzipain competitive ligands. Chemical structures of K777 **(A)**, carvedilol **(B)** and B95 **(C)** generated by using Chemdraw V19.0 (Perkin Elmer Informatics). Cruzipain bound to K777 (PDB entry 2OZ2) was used as a model for our virtual screening, where carvedilol **(B)** was identified as a hit. It is evident the resemblance between carvedilol and B95, which has been crystallized bound to the active site of cruzipain (PDB entry 3KKU).

Of an initial group of 70 structural matches, only carvedilol completely inhibited parasite growth during treatment in the preliminary experiments. Having a promising hit compound, we further characterized its efficacy against *T. cruzi* infection *in vitro* and *in vivo* and its mechanism of action. Despite the lack of evident similarities, ROCS identified carvedilol as a compound related to K777 (structures shown in **Figures 1A, B**). Other common physicochemical properties can be found for both compounds –such as the number H-bond of acceptor groups (5), number of H-bond donor groups (3 & 2), XLogP3 (4.1 & 4.2), RBC (11 & 10) and polar surface <110 Å2 (107 & 75), for K777 and carvedilol respectively. It worth mention that other molecules with high similarity to carvedilol have been described as Cz inhibitors, i.e. B95 (**Figure 1C**, described as compound 27 in (Ferreira et al., 2010). Further analysis showed that carvedilol displayed low inhibitory action on Cz *in vitro* (data not shown).

On the one hand, it has been shown that carvedilol can alter autophagy (Meng et al., 2018; Wong et al., 2018). On the other hand, we previously reported that autophagy is involved in Cz activation (Losinno et al., 2020). Now, we decided to explore the effect of carvedilol on *T. cruzi* autophagy.

### Carvedilol Inhibits the Autophagy Flux in *T. cruzi*

**Figure 2:**
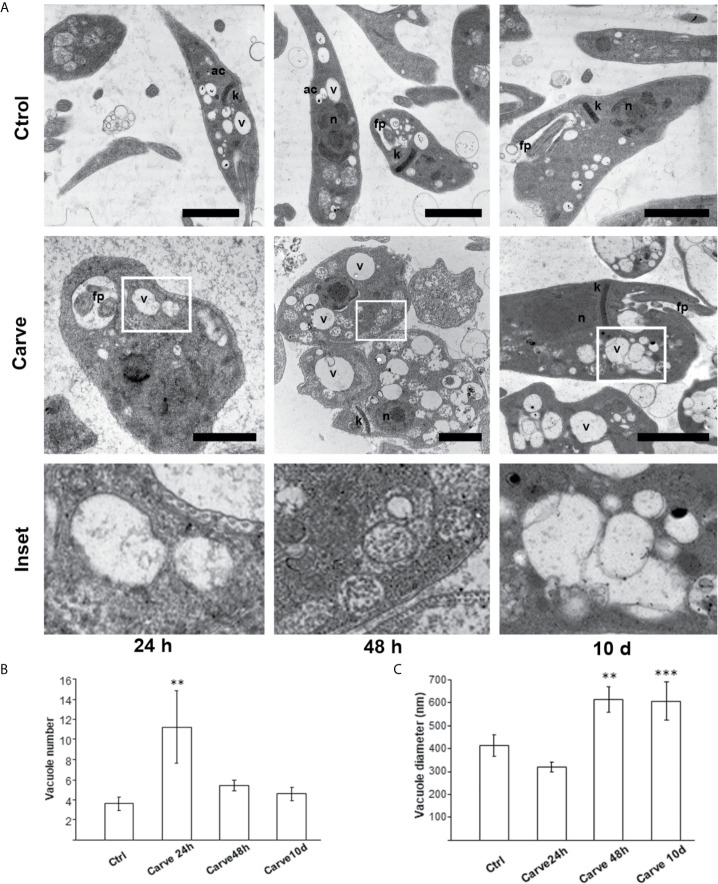
Phenotypic alterations suffered by *T. cruzi* epimastigotes under carvedilol treatment. **(A)** TEM images of control parasites (Ctr) showing the typical elongated body, with terminal flagellum (f) emerging from the flagellar pocket (fp) and normal morphology of reservosomes (r), acidocalcisomes (ac), nucleus (n) and kinetoplast (k); carvedilol treated parasites (Carve) displayed abnormal vacuolization (v) and accumulation of multivesicular structures at 24 and 48 h and after 10 days of treatment. Bars: 2 μm. Insets Insets show the magnifications (5x) delimited in the original photo. Number **(B)** and diameter **(C)** of vacuoles quantified from the TEM images obtained from epimastigotes incubated with DMSO (Ctr) or 10 μM carvedilol (Carve) at the indicated times. Data are shown as mean +/- standard error of 3 independent experiments. **p < 0.01, ***p < 0.001 (Tukey test). A total of 50 cells per group was counted.

Previous reports were contradictory regarding the effect of carvedilol on autophagy. In liver fibrosis, carvedilol suppressed autophagy and promoted apoptosis (Meng et al., 2018), while it was repurposed as an autophagy inducer with beneficial effects on inflammatory diseases (Wong et al., 2018) and after acute myocardial infarction (Zhang et al., 2009). To study the possible effects of carvedilol on *T. cruzi*, we initially analyzed the morphological changes suffered by the parasite cell at the ultrastructural level. We incubated axenic cultures of epimastigotes in the presence of 10 μM of carvedilol at different times (24 h, 48 h and 10 days) and then fixed and processed the parasites for transmission electron microscopy (TEM) analysis. The concentration of carvedilol was selected according to the previous reports (Meng et al., 2018). As shown in the **Figure 2A**, in contrast to control medium, parasites exposed to carvedilol displayed numerous vacuoles with internal vesicles and juxtaposed membranes distributed within the cell body. Multi-lamellar and multi-vesicular structures resembling autophagy compartments were frequently observed inside these vesicles mainly after longer incubation (10 days). Indeed, in some cases cell shape was totally lost and parasites are observed as rounded cells plenty of vacuoles which remind cell death with autophagy phenotype (Levine and Kroemer, 2008) (**Supplementary Figure 1**).

**Figure 3:**
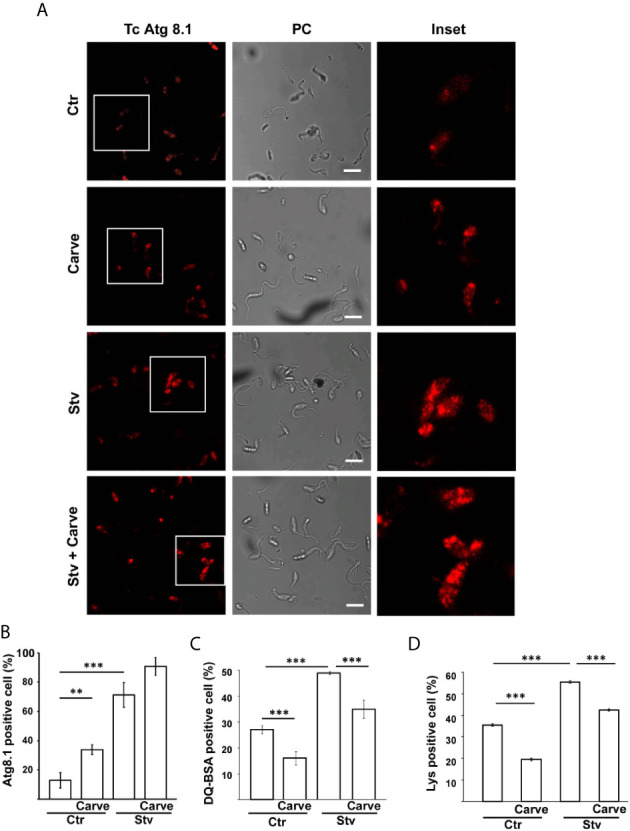
Carvedilol is an autophagy flux inhibitor on *T. cruzi*. *T. cruzi* epimastigotes (Y-GFP strain) were incubated under control (Ctr) or starvation medium (Stv) in the absence (DMSO) or the presence of 10 μM carvedilol for 24 h and then processed for confocal microscopy. **(A)** Detection of the TcAtg8.1 protein by IIF using a specific antibody. Confocal images depict autophagosomes labeled in red (TcAtg8.1) and the phase contrast (PC) for each condition. Scale bar: 10 μm. Insets show the magnifications delimited in the original photo. **(B)** Percentage of parasites with more than two Atg8.1 positive vesicles under each condition. Data shown represent the mean +/- SE from 3 independent experiments. **p < 0.01, ***p < 0.001 (Tukey test). A total of 100 parasites per group was counted. Detection of hydrolytic **(C)** and acidic **(D)** compartments were performed by incubation with DQ-BSA and Lysotracker probes respectively and analyzed by *in vivo* confocal studies. Graphics represent the percentage of parasites positive for DQ-BSA or Lysotracker staining in each condition. Data shown represent the mean +/- SE from 3 independent experiments. ***p < 0.001 (Tukey test). A total of 100 parasites per group was counted.

We quantified the number and size of the vacuoles present at the different times of treatment. Interestingly, the number of vacuoles at 24 h almost tripled compared with control parasites (11.2 ± 3.5 vacuoles in treated parasites against 3.6 ± 0.6 vacuoles in controls) and decreased at later time points (5.4 ± 0.5 and 4.6 ± 0.7 at 48 h and 10 d respectively). In contrast, the size of the vacuoles was similar to control at 24 h (413 ± 46 nm in controls and 318 ± 21 nm under carvedilol treatment) but increased significantly at ≈ 600 nm at 48 h and 10 days (**Figures 2B, C**). To confirm the identity of these vacuoles we labeled the TcAtg8.1 protein, the autophagosome marker of *T. cruzi* (Alvarez et al., 2008; Vanrell et al., 2017), by indirect immunofluorescence. As shown in the **Figure 3A**, TcAtg8.1 positive structures were observed in all conditions, mainly in epimastigotes subjected to starvation medium and carvedilol treatment. Quantification showed that carvedilol increased the percentage of Atg8.1 positive cells from 14.1 ± 5.7% to 34.4 ± 3.2% at control conditions. As expected, starved parasites displayed high numbers of autophagosomes due to autophagy induction, while the addition of carvedilol to the starvation medium caused even higher numbers (71.3 ± 8.5% in starvation medium and 90.6 ± 6.0% in carvedilol plus starvation medium) (**Figure 3B**). Taken together, electron and fluorescence microscopy analysis showed that carvedilol increased the number of autophagosomes in the parasites but, considering the presence of non-degraded materials inside the vacuoles and their large size as observed by TEM, this effect seems to be produced by the accumulation of immature autophagosomes rather than formation of new ones. To investigate this phenomenon, we studied the acidic and hydrolytic properties of the autophagy vacuoles observed under carvedilol treatment, by using the Lysotracker and DQ-BSA probes respectively, following established procedures (Losinno et al., 2020) (**Supplementary Figure 2**). Interestingly, the percentage of cells positive for DQ-BSA, a marker of active hydrolytic compartments was significantly reduced in the presence of carvedilol in both control (from 27 ± 1.5% to 16 ± 2.5%) or starved conditions (from 49.0 ± 0.5% to 35.0 ± 3.5%) (**Figure 3C**). Similarly, carvedilol treatment affected the acidity of these compartments as shown by the minor percentage of cells stained by Lysotracker in these conditions (from 38.2 ± 1.2% to 27.8 ± 0.5% in control medium and from 53.8 ± 1.7% to 42.4 ± 1.1% in starvation medium) (**Figure 3D**), Together, these data indicate that carvedilol inhibited autophagy by inhibiting the autophagy flux, resulting in the accumulation of immature autophagy vacuoles with reduced acidity and hydrolytic activity, which were observed as large vacuoles with non-degraded materials in the TEM images.

### Carvedilol Affects *T. cruzi* Replication and Infectivity

**Figure 4:**
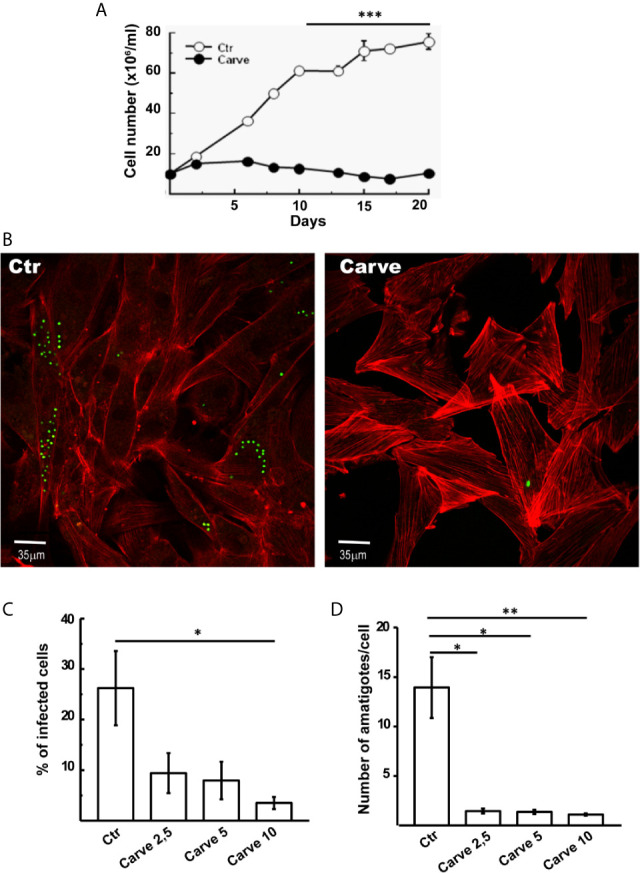
Antiparasitic effect of carvedilol in epimastigotes and amastigotes of T. cruzi. **(A)** Epimastigotes (10x10^6^ parasites/ml) of *T. cruzi* Y-GFP strain were incubated in the absence (DMSO, Ctr) or the presence of 10 μM carvedilol (Carve) at the indicated times. Data shown represent the mean +/- SE from 3 independent experiments. ***p < 0.001 (t-test). H9C2 cells were infected with trypomastigotes of *T. cruzi* Y-GFP strain (MOI=10) for 24 h followed by a chase of 48 h in the presence (Carve) or the absence (Ctr) of carvedilol at different concentrations (2.5, 5 and 10 μM). After fixation, cells were prepared for microscopic studies. **(B)** Confocal images of GFP-expressing amastigotes multiplying within the host cells 48 h after treatment with 10 μM carvedilol versus control (DMSO). H9C2 cells actin myofibrils were stained with rhodamine-phalloidine probe (red). Percentage of infected cells **(C)** and number of amastigotes/cell **(D)** at the different concentrations of carvedilol versus control. Data are shown as mean +/- standard error of 3 independent experiments. *p < 0.05, **p < 0.01 (Tukey test). A total of 100 cells per group was counted.

Given that autophagy is a key process required to maintain cell survival during nutrient deficiency and in vital processes such as cell growth, we next analyzed the effect of carvedilol on *T. cruzi* at different stages of its life cycle. In axenic cultures of epimastigotes, the addition of 10 μM of carvedilol completely abrogated the growth of the *T. cruzi* Y-GFP strain (a strain that express GFP-H2 histone). While control epimastigotes reached the stationary phase (7×10^7^ cells/ml) at day 15, treated parasites remained at the seed concentration (1×10^7^ cells/ml) and below in the same period (**Figure 4A**). To test replication of amastigotes we infected cultures of rat myoblasts (H9C2 cell line) with trypomastigotes of *T. cruzi* Y-GFP (MOI=10) for 24 h, washed and treated with carvedilol at 2.5, 5 and 10 μM for another 24 h. After fixation, cells were stained with TRITC-phalloidine to detect actin filaments and visualize the cell shape and limits, while parasites were directly visualized by the presence of GFP, as demonstrated previously (Vanrell et al., 2017). As shown in the confocal images of **Figure 4B**, several control cells contained numerous amastigotes while very low parasite loads were observed in cells treated with 10 μM carvedilol. Quantitative image analysis showed that carvedilol reduced the percentage of infected cells from 26.1 ± 8.2% in controls to around 10% at 2,5 and 5 μM and even to 4% at 10 μM (**Figure 4C**). Number of amastigotes/cell was also significantly reduced at all three concentrations (from 13.9 ± 3.0 amastigotes in controls to 1.45 ± 0.2, 1.37 ± 0.23 and 1.11 ± 0.1 parasites at 2,5, 5 and 10 μM carvedilol respectively) (**Figure 4D**). Further studies using Alamar blue vital staining showed that carvedilol do not have a toxic effect on host cells at 2,5, 5 or 10 μM (**Supplementary Figure 3**). Therefore, this compound impaired *T. cruzi* replication as axenic forms and as intracellular amastigotes, the latter result validating the repurposed drug as lead for Chagas disease treatment.

**Figure 5:**
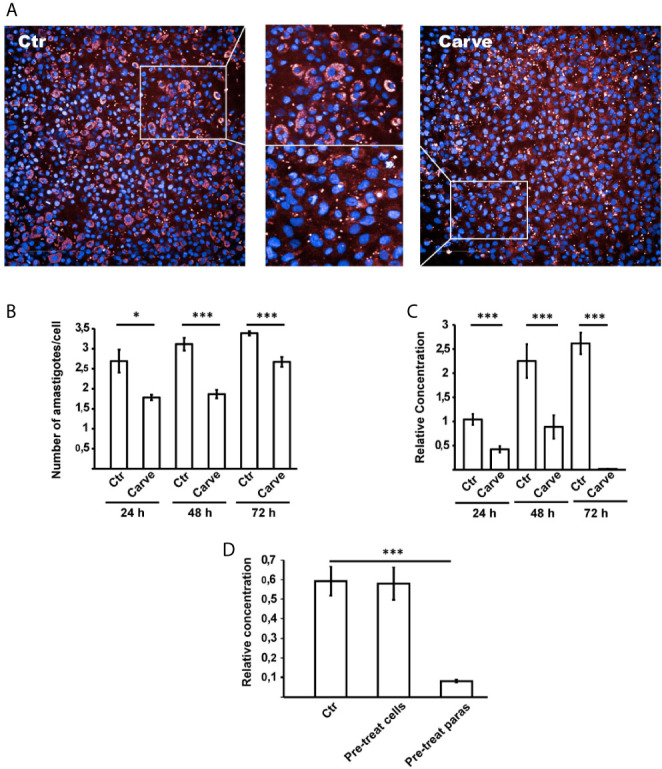
Opera Phenix system and qPCR analysis confirmed action of carvedilol in vitro. HG39 cells were infected with trypomastigotes of *T. cruzi* Tulahuen strain (MOI=10) for 24 hours and treated with 10 μM carvedilol for 24, 48 and 72 h (Carve) or DMSO (Ctr). **(A)** confocal images of each condition taken by the system. **(B)** Number of amastigotes/cell in each group at the indicated times. Data are shown as mean +/- standard error of 3 independent experiments. *p < 0.05, ***p < 0.001 (Tukey test). A total of 100 cells per group was counted. **(C)** Relative concentration of *T. cruzi* DNA in each condition detected by dual-labeled qPCR. Data are shown as mean +/- standard error of 3 independent experiments. ***p < 0.001 (Tukey test). **(D)** Parasite load of HG39 cells infected for 24 h with trypomastigotes of *T. cruzi* Tulahuen strain (MOI=10) previously treated with 10 μM carvedilol for 24 h were compared to cells infected in control conditions (DMSO) or cells pre-treated with 10 μM carvedilol before infection with *T. cruzi* Tulahuen strain (MOI=10). Data are shown as mean +/- standard error of 3 independent experiments. ***p < 0.001 (Tukey test).

We extended our analysis using the Opera Phenix System (PerkinElmer) combining fluorescence microscopy and high-throughput, phenotypic testing of the compound. Human glioblastoma cells (HG39) were infected with *T. cruzi* Tulahuen strain (MOI = 10) for 24 hours. After washing, cells were treated with carvedilol (10 μM) for 24, 48 and 72 h, renewing drug-containing medium every 24 h before fixation. Parasite cytoplasm was labeled with anti-HSP90 antibody by indirect immunofluorescence while host cell and parasite DNA were stained with DAPI. The **Figure 5A** depicts two panoramic images (± carvedilol) taken by the Opera Phenix system and a zoom where it is possible to observe the different levels of infection displayed at each condition. In agreement with previous data, the more robust automatic screening of infected cells showed that the number of amastigotes/cell was significantly reduced at the three time points studied (2.69 ± 0.28 against 1.78 ± 0.06 at 24 h; 3.11 ± 0.15 against 1.86 ± 0.10 at 48 h and 3.38 ± 0.05 against 2.66 ± 0.12 at 72 h) (**Figure 5B**).

We also analyzed the parasite load of infected and treated HG39 cells by dual-labeled qPCR technique (Bifeld et al., 2016) and confirmed a significant drop of the relative concentration of *T. cruzi* DNA in the presence of carvedilol at each time point compared with the host cell DNA which increased its concentration over time due to cell proliferation (relative concentration of DNA: 1.0 ± 0.11, 2.25 ± 0.34, and 2.61 ± 0.22 in untreated cells at 24, 48 and 72 h respectively against 0.42 ± 0.06, 0.88 ± 0.24 and 0.01 ± 0.001 in carvedilol treated cells at the same times) (**Figure 5C**).

In another set of experiments, we studied the effect of carvedilol on trypomastigotes prior to infection. Parasites and host cells were pre-treated with carvedilol 10 μM for 24 h and then combined for an infection for 24 h in control conditions. Parasite loads were evaluated by qPCR. Our data show that pre-treatment of trypomastigotes with carvedilol significantly impaired the infection of host cells in comparison to control parasites (relative concentration of DNA: 0.59 ± 0.07 in control cells against 0.08 ± 0.008 in cells infected with pre-treated parasites) (**Figure 5D**). In contrast, host cells pre-treated with carvedilol did not display differences compared with controls (relative concentration of DNA: 0.58 ± 0.08), indicating that carvedilol activity affects *T. cruzi* but not host cells. Both methodologies confirmed our previous results and highlight the specific activity of carvedilol as an anti-*T. cruzi* drug.

**Figure 6:**
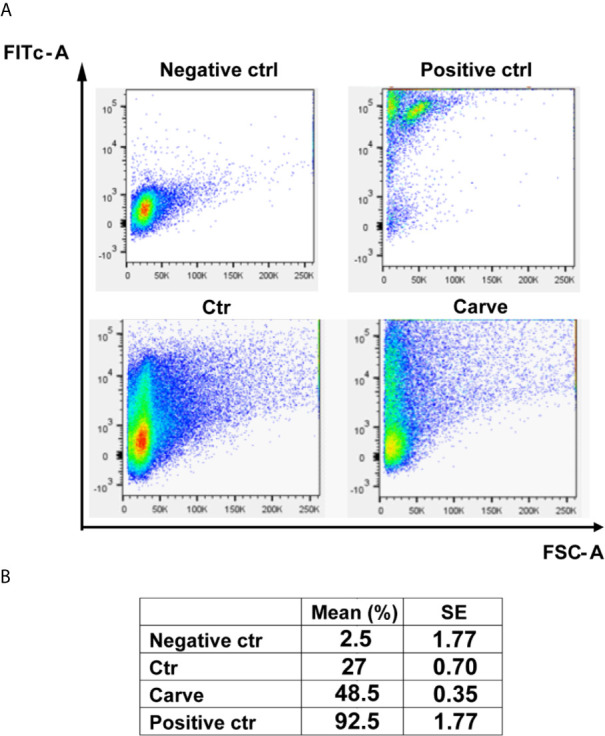
Carvedilol has a trypanocidal effect on *T*. *cruzi* trypomastigotes. Trypomastigotes of *T. cruzi* Tulahuen strain were incubated in the absence (DMSO, Ctr) or the presence of 10 μM carvedilol for 24 h followed by Tunel assay for flow cytometry. Other samples of parasites were treated with DNAse or heated to 90°C for 5 minutes and used as negative and positive controls respectively. **(A)** Graphs depict the 2D plot of each condition. **(B)** Percentage of apoptosis-like process calculated from the data obtained in **(A)** Data are shown as mean +/- standard error of 2 independent experiments.

Considering that many autophagy flux inhibitors induce apoptotic cell death in major eukaryotes, we decided to study treated trypomastigotes for possible apoptosis-like process using a TUNEL assay kit for flow cytometry. Graphics in **Figure 6** showed the number of events distributed according to the size and fluorescence level of cells in each condition. Parasites treated with DNAse or heated at 95°C for 5 min were used as negative or positive controls of apoptosis-like process respectively. Data in the table showed an increment of 27 ± 0.70% to 48.5 ± 0.35% in the number of events detected in the left superior quadrant, indicative of apoptosis-like processes, after 24 h of treatment (**Figure 6**). Control values were 92.5 ± 1.77% for heated parasites (positive control) and 2.5 ± 1.77% for the negative control. Further experiments using axenic cultures of epimastigotes or intracellular amastigotes showed that carvedilol effect were partially reversible and that some parasites resumed growth after drug elimination, although to a small degree (**Supplementary Figure 4**). Jointly, these results indicate that carvedilol has a trypanocidal effect although a small number of parasites can survive at the tested concentrations.

### *In Vivo* Effect of Carvedilol

**Figure 7:**
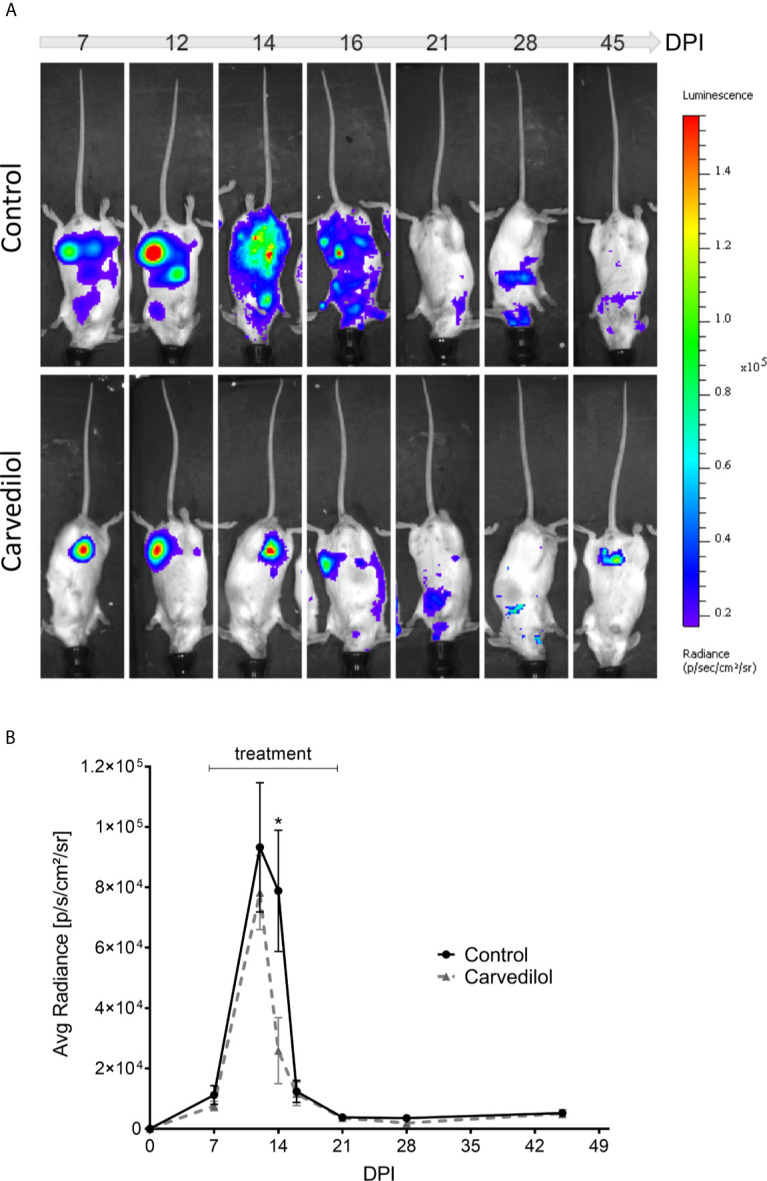
Live imaging of bioluminescent parasites shows action of carvedilol in vivo. 2 groups of 5 mice were infected with 5000 trypomastigotes Tulahuen strain with the Luc-mNeonGreen incorporated. At 7 DPI, 2 animals from each group were treated with carvedilol 25 mg/kg/day for a period of 14 days and periodically measure their body luminescence (see materials and methods). **(A)** Images show the level of parasitism detected by the live imaging luminescence representative of the 2 groups at different days after infection. Right panel indicates the luminescence scale. **(B)** The scatter curve graph shows the means ± SD of radiance measurements obtained in each group at the different days after infection. One-way ANOVA statistical analysis was performed with Tukey multiple comparison test. Significance levels established by p-values ​​*≤0.05.

To confirm the *in vitro* results in a more complex system we studied if the anti-*T. cruzi* action of carvedilol produced beneficial effects in a mice model of infection. To test this, BALB/c mice were infected with 5000 trypomastigotes of *T. cruzi* Tulahuen strain by IP injection. Carvedilol was administered at 25 mg/kg/day once a day from 7-day post-infection (7 DPI) to 45 DPI whereas controls were injected with vehicle during the same time. Carvedilol dose was selected according to the guidance published by the FDA in 2005 for converting doses used in humans to use in mice (FDA/CDER, July 2005) and also by considering previously reports of carvedilol treatments in rodents (Yaoita et al., 2002; Wong et al., 2018; Mohammed et al., 2019). Progression of infection was followed by bioluminescence imaging of luciferase-expressing parasites by subjecting the anesthetized animals to IP injection of luciferin sustractum (Hyland et al., 2008). As shown in the **Figure 7A**, maximal signaling and distribution of parasites in tissues were observed between 12 and 16 DPI in concordance with the average radiance emitted by each group (**Figure 7B**). After 20 DPI, infection was maintained at low levels and no differences were observed between groups suggesting evolution of infection to a chronic stage. Statistical analysis of the radiance emitted through the time for each group showed that a significant reduction in the infection was observed in the carvedilol treated animals at 14 DPI compared to control animals (78830 ± 20120 p/s/cm^2^/sr in animals injected with vehicle *versus* 25890 ± 10970 p/s/cm^2^/sr in mice treated with carvedilol). These data demonstrate that carvedilol also has a positive effect against the *T. cruzi* infection *in vivo* and point it as a possible anti-*T cruzi* drug. More studies are required to confirm these results.

## Discussion

One of the most promising strategies in the search of new treatment options for diseases is drug repurposing, finding second medical uses for established medications. This approach is mainly useful for neglected diseases where drug research and development by private and governmental stakeholders is limited. For Chagas disease, there are currently several repurposed drugs under study. Targets of these drugs are components of the cellular membranes, the trypanothione and redox metabolism, the calcium or pyrophosphate metabolism, the protein and purine synthesis and virulence factors such as cruzipain (Sales et al., 2017). Cruzipain is the major cysteine protease in *T. cruzi*, which plays several roles throughout the parasite life cycle, including parasite energy metabolism, invasion of mammalian cells and host immune evasion (Engel et al., 1998; Vernal et al., 2001; Doyle et al., 2011). Several classes of cruzipain inhibitors have demonstrated effectiveness in *in vitro* and *in vivo* models of infection (Franke de Cazzulo et al., 1994; Du et al., 2002; Choe et al., 2005; Brak et al., 2008; Engel et al., 2010; Mott et al., 2010). The most promising drug, the compound K777 is a vinyl sulphone that covalently binds to the active site of cruzipain. This compound was originally developed as an inhibitor of human cathepsin S by scientists at Khepri Pharmaceuticals (Palmer et al., 1995). Although very active in the preclinical studies (Engel et al., 1998; Barr et al., 2005; Doyle et al., 2007), initial clinical trials of K777 were stopped when the drug showed apparent hepatotoxicity. These concerns were not substantiated by further investigations and K777 is currently re-entering clinical trials (Mckerrow et al., 2009; McKerrow, 2018). By an *in silico* search of different drug collections other authors have found novel chemical scaffolds for cruzipain inhibitory action (Álvarez et al., 2017). Indeed, three approved drugs selected by computer-aided drug repurposing, displayed effective action on acute and chronic murine infection models (Bellera et al., 2015; Sbaraglini et al., 2016). In this study, we performed a ligand-based virtual screening of compounds structurally similar to K777 bound to cruzipain, using the SWEETLEAD database (Novick et al., 2013) and found carvedilol, a drug widely used to treat hypertension and other cardiovascular pathologies. Although spatially similar to K777, carvedilol displayed a weak inhibitory activity on Cz *in vitro* (data not shown) probably due to a minor capacity of carvedilol to establish the key chemical interactions to Cz previously described for vinyl-sulfones (Brinen et al., 2000). In spite of this, we found that carvedilol impaired autophagy flux and, consequently, affected parasite replication and survival.

In our recently published work, we demonstrated that parasite autophagy contributes to the regulation of cruzipain activity during both *T. cruzi* metacyclogenesis and host cell infection (Losinno et al., 2020). Since starvation increases cruzipain activity promoting the acidification and maturation of cruzipain-containing compartments, we suspected that carvedilol may disrupt the fine tuning of pH required for proteolytic activity *in situ*. This was confirmed by a reduction of ≈ 20% in the cells positive for Lysotracker labelling in the parasites treated with carvedilol in both control or starved conditions (**Figure 3D**). Other authors also showed that the effect of carvedilol on pH is similar to chloroquine, a recognized lysosomotropic drug, and that 10 μM of this drug can increase the pH of lysosomes from ≈5.3 to ≈6.5 in a model of hepatic stellate cells (Meng et al., 2018). Total protease activity measured by the self-quenched bovine serum albumin assay, was also reduced (≈ 15%) in the presence of carvedilol (**Figure 3C**), indicating that this compound affects the hydrolytic capacity of *T. cruzi* lysosomal compartments, *i.e.*, lysosomes and autolysosomes. Due to Cz is the most important protease of *T. cruzi*, its activity should be indirectly impaired by carvedilol. Cellular studies *in situ* will be required to confirm this hypothesis.

As a degradative pathway for intracellular materials, autophagy, is a known pro-survival process. Basal autophagy provides protein and organelle quality control required to the normal regulation of cell growth and development. Reduction of autophagy in physiologic and pathologic conditions such as ageing organisms or inflammatory diseases causes inhibition of growth and cell death by apoptosis or other mechanisms (Aguilera et al., 2018; Wang et al., 2019). This seems to be similar for *T. cruzi*, our data from high-throughput microscopy analysis and the highly sensitive dual-labeled qPCR quantification (**Figures 4** and **5**) showed that replication of epimastigotes and amastigotes was significantly impaired under carvedilol treatment, However, when drug is removed, both epimastigotes and amastigotes returned to growth albeit with lower rates compared to controls (**Supplementary Figure 4**). Since our experiments were done with polyclonal cultures of parasites, it is possible that a subpopulation may possess higher tolerance to carvedilol and account for the outgrowth after drug removal. Strains naturally resistant to benznidazole were also previously demonstrated (Campos et al., 2017). Another explanation is the possible generation of quiescent parasites, a process that was previously observed in the replicative stages of *T. cruzi* in the presence of trypanocidal drugs (Sánchez-Valdéz et al., 2018). These observations are evidence that multiple factors can be involved in the success or failure of an anti-*T. cruzi* drug.

Interestingly, in our model of mice infection, a significant reduction (3 time less) of the parasite load was obtained at the peak of infection in mice treated with carvedilol at 25 mg/kg/day in comparison with non-treated animals (**Figure 7B**). In spite of the evolution of both groups to a chronic infection, the lower degree of parasitism during the acute phase of infection in carvedilol treated mice could result in a reduction of cardiac fibrosis in the chronic phase, a process that was previously demonstrated with BNZ treatment. (Francisco et al., 2018). More studies will be done to confirm the efficacy of carvedilol *in vivo* and to propose this compound as an anti-*T cruzi* drug lead with potential for the treatment of human infection.

As an approved drug, carvedilol has an interesting clinical profile that may be advantageous for Chagas patients. It is widely used to treat heart disease and cardiovascular disorders such as hypertension. With exception of the presence of bradycardia, where the β-blockers are contraindicated, other clinical manifestations of Chagas such as arrhythmias, structural heart diseases, cardiomyopathies and cardiac insufficiency may benefit from carvedilol treatment. Its anti-inflammatory effect on the heart (Yuan et al., 2004) may also prevent the fibrosis observed in the cardiac tissue of chronic Chagas patients. Indeed, a randomized clinical trial using renin-angiotensin system inhibitors with the subsequent addition of carvedilol was shown to be safe and associated with benefits in cardiac function and clinical status in patients with chronic Chagas cardiomyopathy (Botoni et al., 2007). These clinical effects in addition to its low price and good availability make this drug a good candidate for Chagas disease treatment in the future.

## Data Availability Statement

The raw data supporting the conclusions of this article will be made available by the authors, without undue reservation.

## Ethics Statement

The animal study was reviewed and approved by Cedars-Sinai Medical Center animal care and use committee (ACUC007053). Los Angeles, California, USA.

## Author Contributions

CVR, SJM, JAC, BNS, MCV, XL and CAL made the experiments, PN and LMP performed the virtual screening and selection of compounds, CVR, SJM, JAC, MCV and LMP made the figures, and performed the statistical analysis. DME, JC and PSR contributed to design the experiments, CVR, MCV and PSR wrote the first draft of the manuscript.

## Funding

Work in this area has been partly supported by grants from Agencia Nacional de Promoción Científica y Tecnológica (PICT# 2013-2757), CONICET (PIP 2014-2016), and Secretaría de Investigación, Internacionales y Posgrado (SIIP, Universidad Nacional de Cuyo) to PR.

## Conflict of Interest

The authors declare that the research was conducted in the absence of any commercial or financial relationships that could be construed as a potential conflict of interest.

## Supplementary Material

Supplementary Figure 1 Dramatic ultrastructural changes observed in carvedilol treated epimastigotes. TEM images of *T. cruzi* parasites in the presence of vehicle **(A)** or 10 μM carvedilol **(B, C)** reveals some parasites lacking the flagellum and with abundant intracellular vesicles which filled the parasite cell after 10 days of treatment. Bars: 2 μm.

Supplementary Figure 2 Hydrolytic and acidic compartments are decreased under carvedilol treatment. *T. cruzi* epimastigotes were incubated under control (Ctr) or starvation medium (Stv) in the absence (DMSO) or the presence of 10 μM carvedilol for 24 h followed by incubation with DQ-BSA and Lysotracker probes in the same conditions at the last 40 min or 2 h respectively. Samples were then prepared for live microscopy. Confocal images depict epimastigotes with DQ-BSA or Lysotracker positive compartments (labeled in red) superposed with the phase contrast images for each condition. Scale bar: 5 μm.

Supplementary Figure 3 Host cell vitality is conserved after carvedilol treatment. Carvedilol (at different concentrations) did not affect mitochondrial activity (cell vitality) measured with Alamar Blue by spectrofluorometry in comparison with cells incubated with DMSO as control.

Supplementary Figure 4 Action of carvedilol is resisted by a low number of epimastigotes and amastigotes. *T. cruzi* epimastigotes were incubated under control (Ctr) or starvation medium (Stv) in the absence (DMSO) or the presence of 10 μM carvedilol **(A)** After 20 days of treatment, epimastigotes were diluted (to 0.2x10^6^ parasites/ml) in fresh medium and the growth was quantified at the indicated times. **(B)** H9C2 cells were infected for 24 h followed by 48 h of treatment in control media (DMSO) or with 10 μM carvedilol (Treatment) and fixed. Other samples were washed and incubated an additional time (48 h) in control media to allow the replication of amastigotes (Recovery). Graph shows the number of amastigotes/cell quantified in each condition.
