# Supplementary figures and images for: Repurposing Carvedilol as a Novel Inhibitor of the Trypanosoma cruzi Autophagy Flux That Affects Parasite Replication and Survival
Source: Front Cell Infect Microbiol. 2021 Aug 12;11:657257. doi: 10.3389/fcimb.2021.657257 (PMC8406938; doi:10.3389/fcimb.2021.657257)

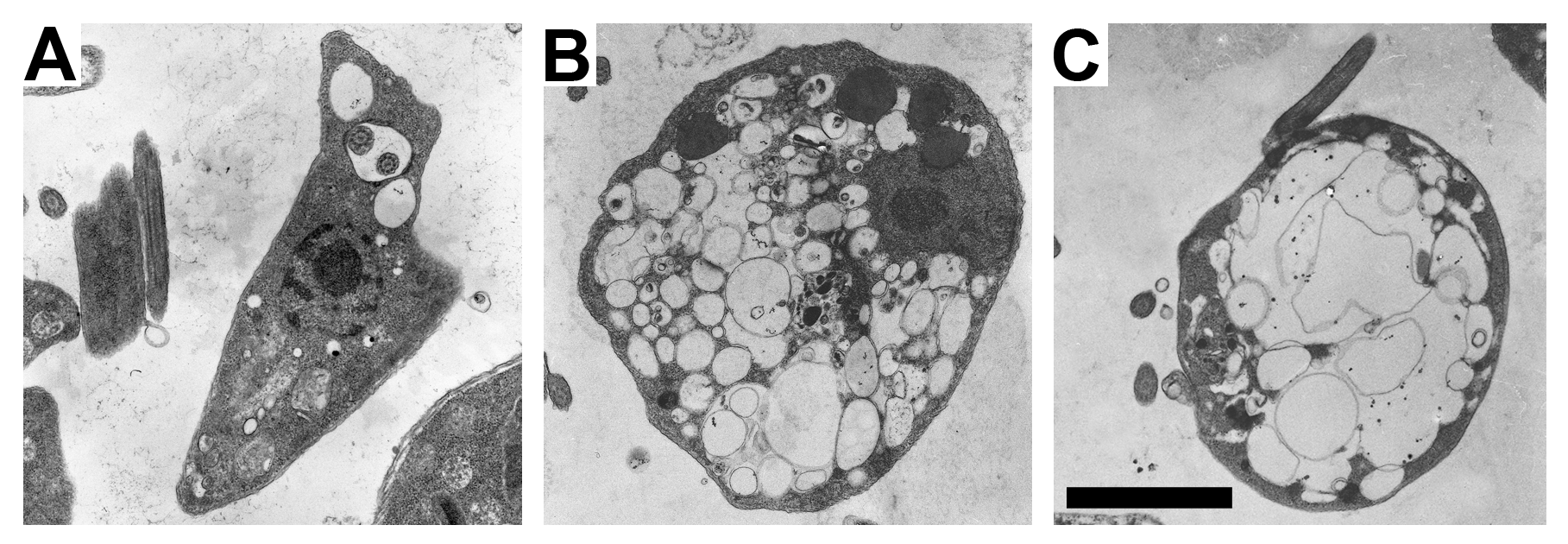

Supplement: Supplementary Figure 1 — Dramatic ultrastructural changes observed in carvedilol treated epimastigotes. TEM images of T. cruzi parasites in the presence of vehicle (A) or 10 μM carvedilol (B, C) reveals some parasites lacking the flagellum and with abundant intracellular vesicles which filled the parasite cell after 10 days of treatment. Bars: 2 μm. [file Image_1.tif]

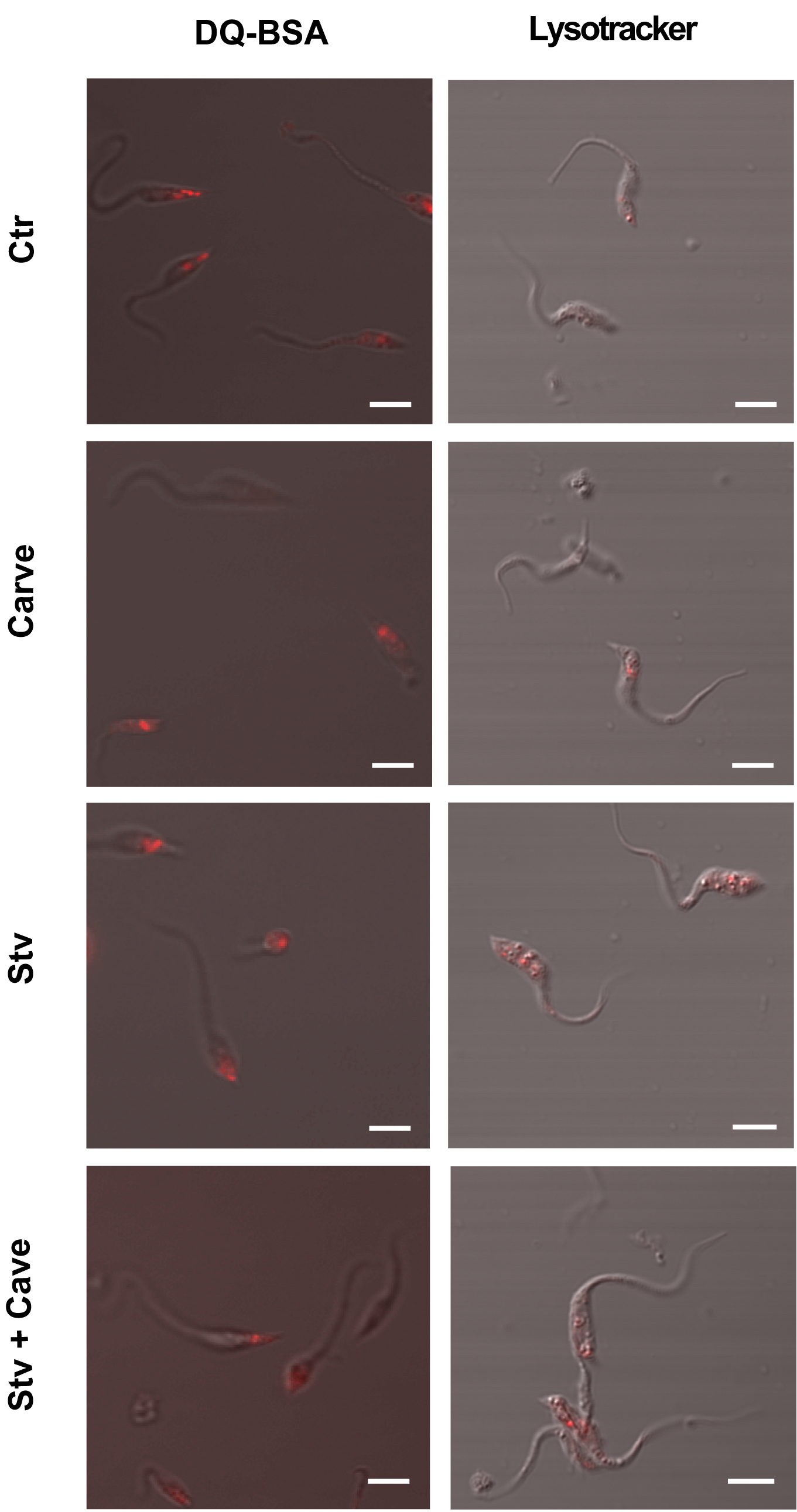

Supplement: Supplementary Figure 2 — Hydrolytic and acidic compartments are decreased under carvedilol treatment. T. cruzi epimastigotes were incubated under control (Ctr) or starvation medium (Stv) in the absence (DMSO) or the presence of 10 μM carvedilol for 24 h followed by incubation with DQ-BSA and Lysotracker probes in the same conditions at the last 40 min or 2 h respectively. Samples were then prepared for live microscopy. Confocal images depict epimastigotes with DQ-BSA or Lysotracker positive compartments (labeled in red) superposed with the phase contrast images for each condition. Scale bar: 5 μm. [file Image_2.tif]

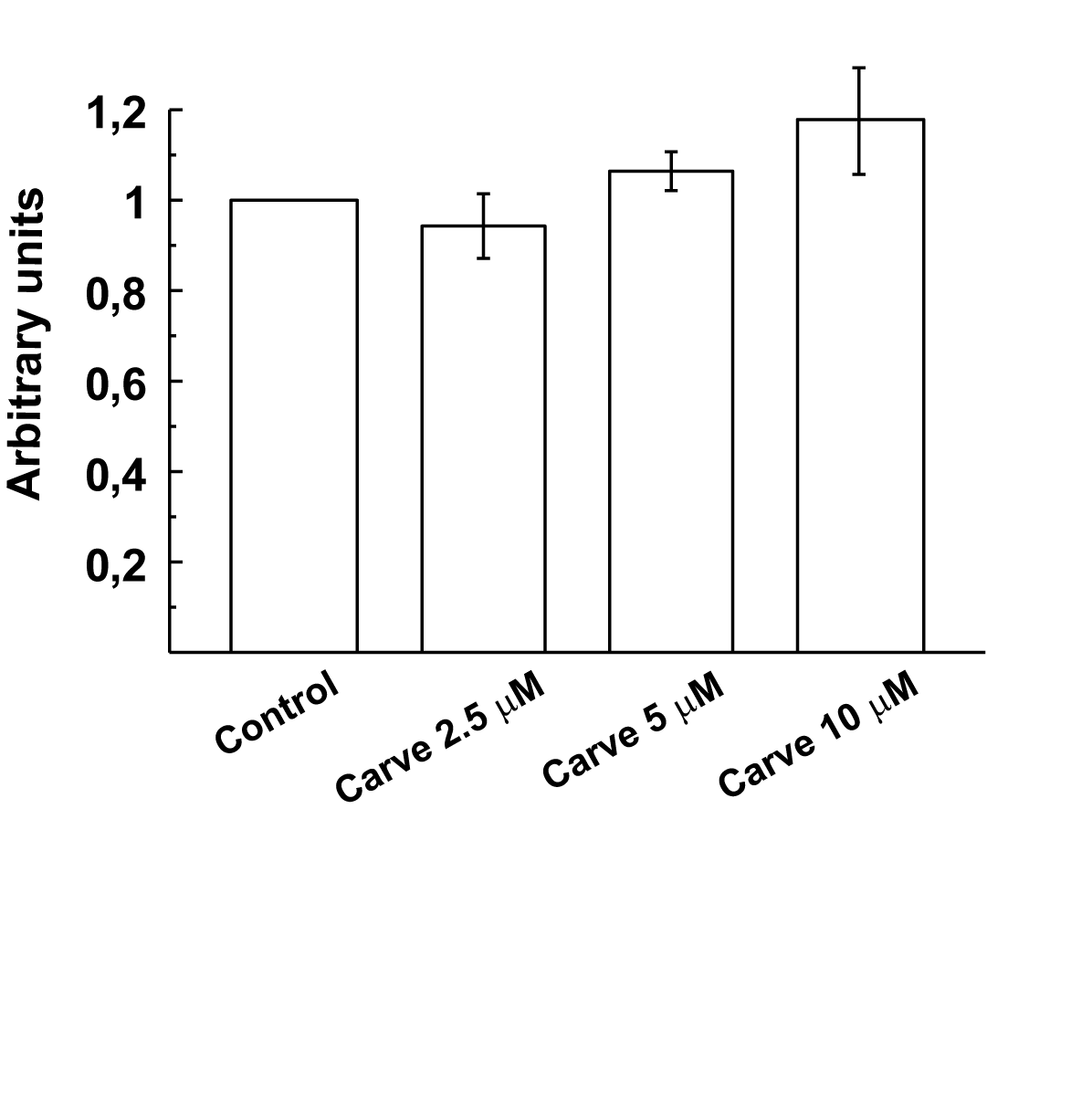

Supplement: Supplementary Figure 3 — Host cell vitality is conserved after carvedilol treatment. Carvedilol (at different concentrations) did not affect mitochondrial activity (cell vitality) measured with Alamar Blue by spectrofluorometry in comparison with cells incubated with DMSO as control. [file Image_3.tif]

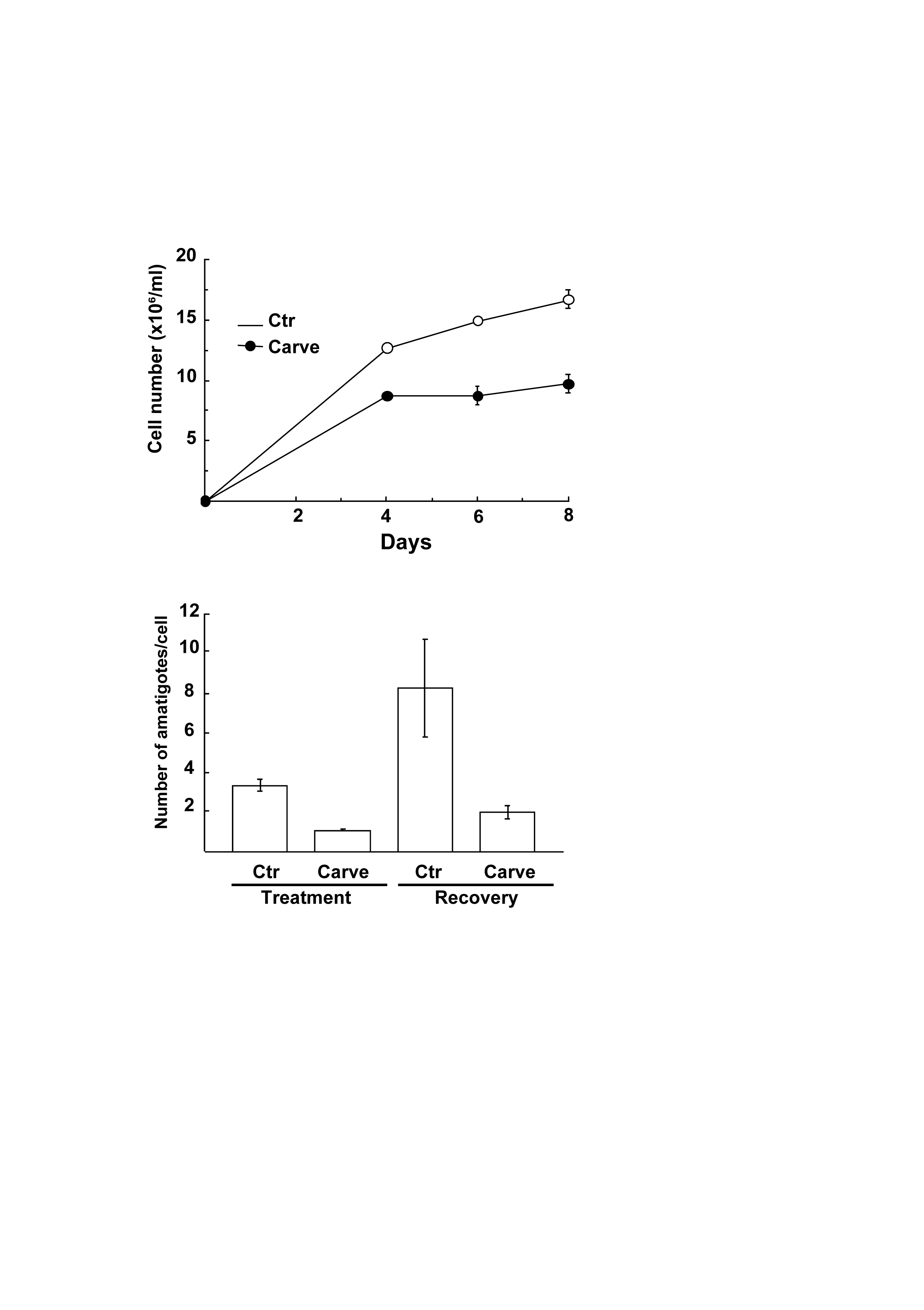

Supplement: Supplementary Figure 4 — Action of carvedilol is resisted by a low number of epimastigotes and amastigotes. T. cruzi epimastigotes were incubated under control (Ctr) or starvation medium (Stv) in the absence (DMSO) or the presence of 10 μM carvedilol (A) After 20 days of treatment, epimastigotes were diluted (to 0.2x10^6^ parasites/ml) in fresh medium and the growth was quantified at the indicated times. (B) H9C2 cells were infected for 24 h followed by 48 h of treatment in control media (DMSO) or with 10 μM carvedilol (Treatment) and fixed. Other samples were washed and incubated an additional time (48 h) in control media to allow the replication of amastigotes (Recovery). Graph shows the number of amastigotes/cell quantified in each condition. [file Image_4.tif]
